# An Analysis of Waiting Time for Emergency Treatment and Optimal Allocation of Nursing Manpower

**DOI:** 10.3390/healthcare10050820

**Published:** 2022-04-28

**Authors:** Pei-Hung Liao, William Chu, Chen-Shie Ho

**Affiliations:** 1 School of Nursing, National Taipei University of Nursing and Health Sciences, Taipei 112, Taiwan; peihung@ntunhs.edu.tw (P.-H.L.); healthai95@gmail.com (W.C.); 2 Department of Orthopedics, Cheng Hsin General Hospital, Taipei 112, Taiwan; 3 Department of Healthcare Administration, Asia Eastern University of Science and Technology, Taipei 112, Taiwan

**Keywords:** waiting time, nursing manpower, optimal allocation

## Abstract

Objective: Emergency care is the frontline of the healthcare system. Taiwanese typically seek emergency care when suffering from an acute or unknown illness, which leads to a large number of emergency patients and the related misallocation of nursing manpower, and the excessive workloads of emergency service providers have become serious issues for Taiwan’s medical institutions. Participants: This study conducted purposive sampling and recruited patients and nursing staffs from the emergency room of a medical center in New Taipei City as the research participants. Methods: This study applied the queueing theory and the derived optimal model to solve the problems of excessive workloads for emergency service providers and misallocation of nursing manpower, in an attempt to provide decision makers with more flexible resource allocation and process improvement suggestions. Results: This study analyzed the causes of emergency service overload and identified solutions for improving nursing manpower utilization. Conclusions: A wait-time model and the queueing theory were used to determine resource parameters for the optimal allocation of patient waiting times and to develop the best model for estimating nursing manpower.

## 1. Introduction

Emergency medical services are an important part of the medical care system and provide indispensable emergency treatments that save lives, shorten the course of diseases, and maintain physical functions. In the context of Chinese culture, most people will choose emergency treatment for emergency cases or for those where the nature of the illness is unknown. Such behavior leads to an increase in the number of emergency visits, which results in emergency care providers being faced with high pressure, excessive patient numbers, and frequent public relations issues and disputes, as well as insufficient beds, human resources, and salaries [1].

The average daily number of emergency cases treated by Taiwanese medical institutions increased from 12,893 in 2006 to 19,807 in 2016, a 45 percent increase [2]. Today, more than 50 percent of emergency rooms are faced with “serious” or “dangerous” degrees of overcrowding, which results in prolonged waiting times for patients and delays in their treatment. The turnover rate among new nurses in Taiwan is around 20 to 30 percent, which is nine percent higher than the rate of other industries, as new nursing staff face multiple stresses due to internal factors or their external environments [3]. That said, nursing errors are not typically caused by internal factors; rather, in a complicated medical system, they are usually the result of external environmental factors, with one of the most critical issues being the design of the overall workflow and the use of auxiliary systems. Relatedly, unfriendly working environments can be a significant source of stress for nurses [4].

As emergency care overcrowding is a topic of great concern to medical institutions, research on emergency care overcrowding has expanded rapidly in recent years [5]. Although clinical staff, managers, and researchers are working to ease emergency care overcrowding, the problem continues to worsen [6]. Emergency care overcrowding can weaken a hospital’s adaptability to changes, which increases the likelihood of medical errors and adverse events and results in delayed treatments and longer waiting times. Relatedly, patients may experience longer hospital stays and increased medical costs due to overcrowding, as well as face higher rates of subsequent visits and even death. Long waiting times are a major source of patients’ medical service-related complaints and a common focus of improvement efforts [7,8]. In fact, the amount of time during which patients are actually treated only accounts for a small part of the entire process, while long waiting times in hospitals are, in general, unavoidable. Emergency care overcrowding seriously affects the quality of service of emergency departments, jeopardizes patient safety, and reduces the morale of medical staff, which can all cause medical disputes. The medical treatment process should be effectively improved, in order that it can become a smooth experience for patients. Furthermore, related services should also be provided, such as information for waiting patients to help them understand and evaluate how long they have to wait, or services with a personal touch to reduce the negative emotions of patients [9]. Thus, it is important for emergency medical treatment providers to analyze the causes of emergency care overcrowding and find solutions to improve the quality of emergency medical care [10].

This study explored how to address the current situation of emergency visits to reduce waiting time [11], sought to understand the five levels of triage, and conducted an analysis based on the queueing theory to optimize waiting times. According to past research models, nursing staff classify the illnesses, and then the doctors directly make their diagnoses and determine medical decisions and care treatments [11,12]. Our system model incorporated feedback on the doctor’s diagnosis in the classification results and performed comparison-based evaluations of consistency to improve the effectiveness of the evaluation model. The main objectives of this study were, as follows: (1) to enable hospitals to capitalize on the queueing model to reduce waiting times; (2) to determine the differences between the queueing model and the actual observations; and (3) to explore nursing manpower and its impact on waiting times for emergency visits by adjusting various parameters. In Taiwan, the front-line evaluators and nursing staff in emergency rooms are all nurses, and there are emergency departments at all levels of the medical institutions due to cultural influence. The public is not familiar with the education required for the emergency medical classification system. However, the calculation method of nursing manpower, based on the total number of patients, has not been adjusted for about 20 years, thus, in recent years, there has been a shortage of manpower in nursing staff around the world. Moreover, frequent clinical overtime and staff to support related units have been used to fill the nursing vacancies, which results in the physical and mental fatigue of nurses, increases medical negligence, and affects patient safety. Therefore, this study further discusses this phenomenon [13].

## 2. Article Review

### 2.1. Emergency Medical Service

In 1994, the Society for Academic Emergency Medicine (SAEM) defined emergency medicine as a medical specialty that involves assessment, handling, treatment, and the medical care system. As part of a national medical safety net, emergency rooms provide all-around 24-h medical care. Hanlon stated that emergency care involves sudden, emergency medical conditions that affect an individual’s safety and health [14]. The emergency department (ED) is the frontline of medical services, as well as the place where patients and their family members immediately think of when they are in urgent need of medical care [15]. The National Health Insurance Administration defines emergency care as medical attention that involves the provision of immediate and appropriate treatments to patients, in order to save their lives, shorten the course of the disease, retain limbs, or maintain the patients’ functions. The standards for the application of emergency medicine (Ministry of Health and Welfare Health Insurance No. 84029441) are, as follows [16]: people with mental illness that endanger the safety of others or themselves; with acute injuries caused by major accidents; with statutory or reportable infectious diseases that require immediate treatment; and those with unstable vital signs or other life-threatening symptoms.

### 2.2. Illness Classification Criteria

Illness classification is meant to classify patients to determine the order of doctor’s visits. The classification system used in Taiwan is based on the Department of Health’s (now known as the Ministry of Health and Welfare) hospital emergency department evaluation criteria, and emergency patients are classified into four levels according to this classification system. In 2006, the Department of Health commissioned the Society of Emergency and Critical Care Medicine, Taiwan, ROC and the Taiwan Association of Critical Care Nurses to set up an illness classification working group to develop a Taiwan Triage Acuity Scale (TTAS), based on the Canadian Triage Acuity Scale (CTAS), and establish a computer-assisted system (eTTAS) to help caregivers make assessments [17]. At present, emergency medicine implements the five-level triage standards, which are based on the patients’ respiratory distress, blood mobility changes, consciousness level, body temperature, pain level, and injuries. The descriptions of the levels are, as follows [18]:

Level 1: Emergency resuscitation: in cases of hemorrhage and unconsciousness from a car accident, first aid must be given immediately;
Level 2: Critical: in cases of bleeding from a car accident, but with stable vital life signs, the recommended treatment and waiting time is 10 min;
Level 3: Urgent: in cases of mild respiratory distress and breathing difficulties, the recommended wait and reassessment time is 30 min;
Level 4: Sub-urgent: the recommended wait and reassessment time is 60 min;
Level 5: Non-urgent: the recommended wait and reassessment time is 120 min.

### 2.3. Problems Pertaining to Emergency Care Waiting Time

The Emergency Department Length of Stay (ED LOS) refers to the time from the patients’ entry into the emergency department, through the classification of the illness, the visit, the inspection, the consultation, the observation, and the wait for treatment, to departure from the emergency department. Patient waiting times in the emergency room are getting longer, and there are many factors that affect the stay of emergency patients. Professor Shengchuan Hu took the lead in the computerized monitoring of emergency room traffic in 1993, and found that the wait for an IV drip, the wait for a consultation, and the wait for special examinations are often the main causes of prolonged stays of emergency patients in the hospital. In 2000, Kuan et al. concluded that the wait for reports is the main reason for extended waiting times. The emergency departments of medical centers witness overcrowding every day, which often creates a high pressure and disaster-prone environment for emergency doctors [19]. From the perspective of the hospital, Forster et al. [18] explored the impact of hospital bed occupancy rates on emergency stay times and patient disposal, and the results of the study showed that the hospital bed occupancy rate significantly affects the stay time of emergency patients [18]. In 2008, Wu et al. studied the factors related to emergency stay time and found that the patients’ stay time was affected by the hospital’s medical inspection process and the patients’ individual traits [9].

The “waiting” problem in hospitals has become increasingly unacceptable to patients in today’s efficient and time-critical society. Ella Jordan [20] pointed out that long waiting times or queues can directly affect the service quality of the hospital, which causes patients to have negative emotions in the process of waiting, and thus, lowers the evaluation of the hospital [4]. Therefore, it is important for hospitals to maintain patient waiting times at a reasonable level if they wish to improve their competitiveness [8].

### 2.4. Nursing Manpower

Emergency nursing staff are on the front line of clinical duties and work as an important safeguard for patient safety. In the current arrangement of nursing staff shifts in Taiwan, only 330 nurses are designated for every 100,000 people, a number that is significantly lower than that in other countries (780 in New Zealand, 730 in the UK, and 500.1 in Singapore) [21,22], thus, clinical nursing staff quit their jobs mostly due to work stress. According to research, junior emergency nursing staff quitting their jobs accounts for 33% of the total, which is much higher than the average of 11% in other careers [23]. The major reasons behind this are a lack of clinical experience and nursing skills, and work overload, which adversely affects their daily life.

Emergency departments usually have large workloads, an imbalance between the ratio of doctors and nurses, and a dependency on nurses’ work, and these problems may exacerbate the lack of communication between doctors and nurses in the clinical environment [24]. However, information technology can change traditional clinical team communication. Studies have shown that the use of asynchronous communication systems and optimized work processes can greatly improve communication between doctors and nurses and nursing team members, thereby improving the quality of health care [25,26].

### 2.5. Queueing Theory

The queueing theory, also known as the waiting theory, mainly studies the characteristics of various waiting systems, such as the average waiting time of each customer, the average number of customers in the system, and system performance parameters, through which the waiting system can be improved [12]. In most waiting systems, customers successively enter the waiting system (i.e., join the waiting line), where the service provided by the facility is accepted according to the queue discipline, and customers leave the waiting system after the service is completed. The queueing theory is included in emergency medicine, where the patients’ average wait time and the average length of the queue are obtained by measuring the patient arrival rate and the medical staff service rate. The waiting system generally consists of four components, including customers, waiting line, waiting rules, and service facilities, which are described, as follows [10]:

- Customer: This component has two characteristics, one of which is the population, i.e., the total number of customers. It is roughly divided into the two categories of a limited group and an unlimited group. The other component is the customer arrival rate, or inter-arrival time, which requires a suitable statistical allocation to describe;
- Waiting line: The main feature is the waiting line capacity, which can be divided into wired and wireless. Since the mode in the wireless case is much easier than that in the wired case, when the capacity of the waiting line is large enough, it is assumed that the waiting line capacity is infinite. In addition, the waiting line capacity plus the number of customers that the service facility can accommodate are equal to the system capacity;
- Waiting rules: The order by which customers receive services, and the most common rule is first come first served (FCFS). There are also waiting rules based on random selection or priority;
- Service facilities: A service agency can contain one or more servants. Facilities that contain more than one servant are called facilities with parallel services. Service facilities have the service rate attribute.

**Figure 1:**
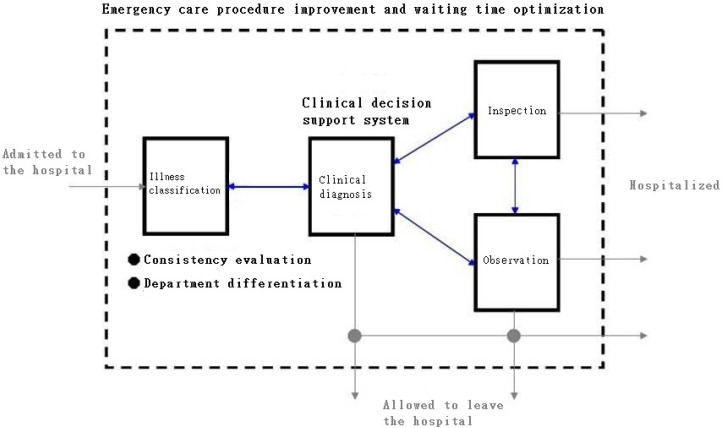
Process flow in the emergency care department.

There are often multiple waiting lines in a real system, which has led to the development of the queueing network model [11]. In the queueing network model, each group of service facilities and their waiting lines are the only node, and according to the relationship of the arrival (or departure) of the customer with the outside world, the queueing network model can be divided into the open and closed categories. As shown in Figure 1, the customer source group in the open network model is infinite, while it is limited in the closed network.

## 3. Methods and Materials

**Figure 2:**
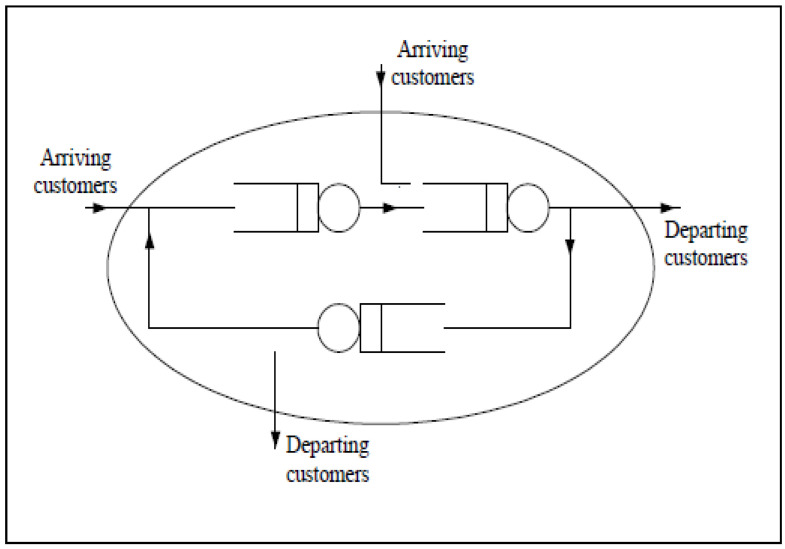
Queueing network model.

**Figure 3:**
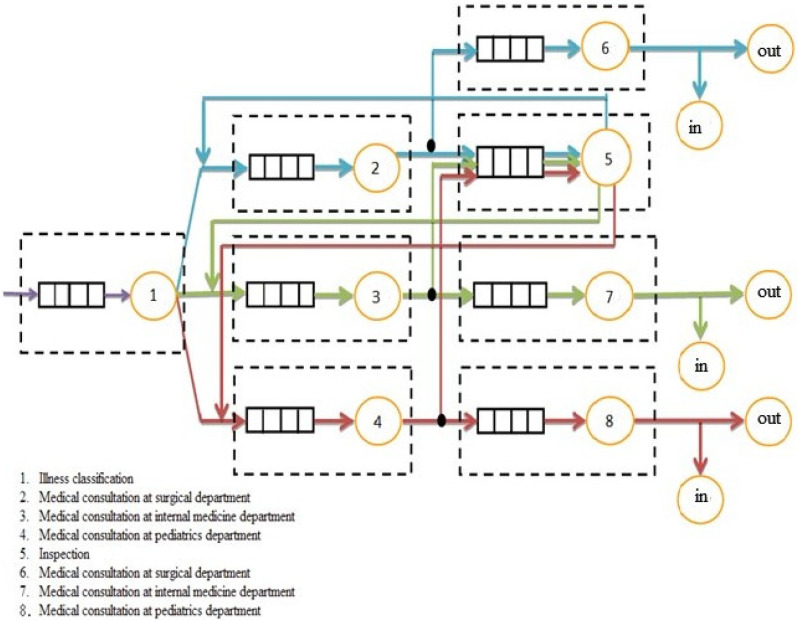
Proposed simulation architecture.

In an attempt to provide decision makers with more flexible resource allocation and process improvement suggestions, this study collected and recorded the patients’ stay times after their illness classification, inspection, diagnosis, consultation, observation, wait for treatment, etc., analyzed the process efficiency by the queueing theory, derived the optimal model to reduce the waiting time for emergency treatments, and determined the effective allocation of nursing manpower. The information for this study came from the medical center, which manually recorded the patients’ waiting times from the start of the emergency examination to the time of leaving the hospital. The tools included SPSS, AMPL/CPLEX, and JMT (Java Modeling Tool). The process flow in the emergency care department, the general queueing network model, and the proposed simulation architecture are depicted in Figure 2 and Figure 3, respectively.

### 3.1. Clinical Trials

This study conducted purposive sampling and recruited patients and nursing staff from the emergency room of a medical center in New Taipei City as the research participants. A total of 201,530 cases were collected between 1 January (starting time: 0:00) and 31 December (ending time: 24:00), 2016, were obtained from the computer database via IRB approval, and three shifts of nursing staff in the emergency department were analyzed.

In addition, derivation was made based on the on-the-scene observations, caregiver manpower, the doctor’s disposal time, the patient flow factors, and the implementation of a special line for mild illness.

The results provides the changes in patient waiting times under different nursing manpower allocations without performing other administrative tasks (Table 1, Table 2, Table 3, Table 4 and Table 5).

#### 3.1.1. The Impact of Long Reported Wait Time on Waiting Time

The change in waiting times caused by each one-minute reduction in reported wait times was, as follows:

#### 3.1.2. The Influence of Nursing Manpower on Waiting Time

The change in waiting times with each additional caregiver was, as follows:

#### 3.1.3. The Effect of the Doctor’s Treatment Time on Waiting Time

The change in waiting times for each one-minute reduction in the doctor’s treatment time was, as follows:

#### 3.1.4. The Effect of Patient Flow on Waiting Time

The results of the burst entry, simulated using Pareto distribution, were as follows:

#### 3.1.5. The Impact of the Implementation of a Special Line for Mild Illness on Wait Times

The effects of different dispersion probabilities were as follows:

## 4. Results

### 4.1. Demographics

1. This study obtained the samples from a medical center in New Taipei City, and the effective sample was 2000 people. In terms of gender, 1150 (57.5%) were male and 850 (42.5%) were female; 1190 (59.5%) belonged to the surgery department, 630 (31.5%) belonged to the internal medicine department, and 180 (9%) belonged to the pediatric department;
2. The proportion of illnesses at all levels was, as follows: The effective sample was 2000 people for the proportion of illnesses at all levels. The first level had 30 people (1.5%), the second level had 210 (10.5%), the third level had 740 (37%), the fourth level had 690 (34.5%), and the fifth level had 330 people (16.5%);
3. Regarding the use of X-rays in each department: In the surgery department, 520 people (43.7%) did not have X-rays, while 670 people (56.3%) had X-rays; in the internal medicine department, 130 people (20.63%) did not have X-rays, while 500 people (79.37%) had X-rays; in the pediatric department, 60 people (33.33%) did not have X-rays, while 120 people (66.67%) had X-rays;
4. Regarding the post-treatment status of patients in each department: There were 620 people (52.1%) in the surgery department who were allowed to leave the hospital, 120 people (10.1%) who were hospitalized, and 450 people (37.82%) who remained under observation; in the internal medicine department, 390 (61.9%) were allowed to leave the hospital, 30 (4.76%) were hospitalized, and 210 (33.33%) remained under observation; in the pediatric department, 150 people (83.33%) were allowed to leave the hospital, 0 (0%) were hospitalized, and 30 (16.67%) remained under observation.

### 4.2. Model Verification Results

After the measured data were analyzed by SPSS, the λ and µ of each station could be known, where λ was the average arrival rate and μ was the average service rate.

- The average arrival rate in Queue 1 (illness classification) was 11.84 person/hour, and the average service rate was 11.84 person/hour, thus, there was no overcrowding;
- The average arrival rate in Queue 2 (internal medical department) was 3.5 person/hour, and the average service rate was 3.5 person/hour, thus, there was no overcrowding;
- The average arrival rate in Queue 3 (surgical department) was 7.2 person/hour, and the average service rate was 7.2 person/hour, thus, there was no overcrowding;
- The average arrival rate in Queue 4 (pediatric department) was 1.14 person/hour, and the average service rate was 1.14 person/hour, thus, there was no overcrowding;
- The average arrival rate in Queue 5 (Internal Medicine) was 3.5 person/hour, the average service rate was 2.25 person/hour, and the average arrival rate was greater than the average service rate, which resulted in overcrowding;
- The average arrival rate in Queue 6 (inspection) was 4.25 person/hour, and the average service rate was 4.25 person/hour, thus, there was no overcrowding;
- The average arrival rate in Queue 7 (surgical care) was 7.2 person/hour, the average service rate was 4.25 person/hour, and the average arrival rate was greater than the average service rate, which resulted in overcrowding;
- The average arrival rate in Queue 8 (pediatric care) was 1.14 person/hour, the average service rate was 0.67 person/hour, and the average arrival rate was greater than the average service rate, which resulted in overcrowding;

The (M/M/1) model results, as obtained through the application of JMgraph software to simulate the queueing model, showed that:

The measured time in Queue 1 (illness classification) was 1 min, the estimated time was 1 min, and the difference was 0%;
The measured time in Queue 2 (internal medical department) was 7.02 min and the estimated time was 8.82 min, with a difference of 20.4%;
The measured time in Queue 3 (surgical department) was 6.47 min and the estimated time was 4.81 min, with a difference of 25.66%;
The measured time in Queue 4 (pediatric department) was 3.78 min and the estimated time was 2.07 min, with a difference of 45.2%;
The measured time in Queue 5 (Internal Medicine) was 16.89 min and the estimated time was 9.27 min, with a difference of 45.1%;
The measured time in Queue 6 (inspection) was 1.4 min and the estimated time was 1.97 min, with a difference of 28.9%;
The measured time in Queue 7 (surgical care) was 18.58 min and the estimated time was 10.97 min, with a difference of 41.33%;
The measured time in Queue 8 (pediatric care) was 10.79 min and the estimated time was 6.61 min, with a difference of 38.7%.

In summary, the average total stay time was 67.21 min, while the estimated average total stay time was 32.56 min, for a difference of 51.55%. This substantial difference may be attributed to measurement errors, the model settings, or the multiplication effect (accumulation of error).

The major causes of emergency service overcrowding, as determined by the best allocation model, included insufficient medical manpower, prolonged waiting times for reports, and failing to allocate manpower according to different diagnoses and treatments. We observed that, during the peak season of enterovirus for children in summer and cardiovascular diseases (such as coronary heart disease and stroke) in winter in Taiwan, there were always sudden increases in the number of emergency service patients, such as when parents would bring their children to hospitals late at night due to high fever. Thus, adjustments in nursing manpower and the relevant examination processes are advised.

## 5. Discussion

### Manpower Allocation of Emergency Care

At present, in non-mass casualty incident modes, the number of nursing staff in an emergency department can be calculated according to the number of beds and the ratio of nurses to patients, such as five specialist nurses, and the number of nurses for each shift is eight, seven, and seven, for the day, evening, and night shifts, respectively. In general, the nursing staff working in an emergency department are dynamically scheduled to support the immediate needs of each site. In order to achieve the best allocation of nursing manpower, this paper proposes the following three strategies:

1. Make adjustments according to the time period. The nursing staff at each site can be flexibly increased during peak periods. The on-site observation results of this survey show that the time period for the highest number of emergency visits is between 18:00 and 20:00, whereas the lowest number occurs between 03:00 and 04:00. The head nurse can flexibly deploy the number of personnel according to the dynamic changes in patients in each part of the day to achieve maximum work efficiency for the existing manpower. Due to the uncertainty of the number of visits by emergency patients, there may be emergencies or batches of patients at any time, especially in the early hours; therefore, reasonable preparation for shifts is particularly important. In addition, nursing staff can be reasonably allocated according to the characteristics of the month. According to the results of this survey, a higher number of emergency patient visits occurred in March, April, July, and August, which may be related to the temperature rise and hot weather. In addition, nursing staff can be reasonably allocated according to the characteristics of different types of workdays;
2. Make adjustments according to the proportion of the number of people in different inspection categories. As patient arrival times in the hospital are not fixed, it is difficult to effectively plan human resources in advance; however, the current number of people in the hospital can be monitored by a sliding time window, and this method can be based on the total number of people in the hospital, or according to the number of different emergency sites. Taking the latter as an example, due to the different resources and processes required for the treatment of patients in different classes, the required nursing human resources should also be adjusted. According to the queueing structure of this study, in order to optimize waiting times, for example, when the proportion of patients in each class is equal, stations 1, 2, 3, and 4 can be allocated with one, two, one, and one nurse, respectively, to perform related nursing operations. However, when the proportion of patients in each class linearly increases, one, one, two, and three nursing staff can be allocated to perform related nursing operations at stations 1, 2, 3, and 4, respectively. The arrangement of the time window can be estimated according to the time distribution of patient arrivals, and then be adjusted, according to the actual progress of the medical treatment;
3. Make adjustments according to the waiting time at each station. As the clinical path of each patient is different, the general medical process will cause larger waiting times for patients with more repeated processes. Due to the variable number and repetition of processes, it is difficult to make predictions in advance; therefore, the assigned manpower can be dynamically adjusted according to the waiting time of each site. The waiting time of each station can be calculated based on the waiting time of a single station, or the accumulated waiting time starting from triage. The calculation basis can also be divided into individual or overall waiting times, in order to determine the nursing manpower required by each site. In general, site manpower allocation can be carried out according to the personal characteristics of the nursing staff on duty that day, as well as the nature of the business content of each site. It is worth mentioning that this manpower allocation method is completely dynamic; that is, the human resources of each station are completely dependent on the current patient waiting times. At the same time, regardless of the dynamic adjustment method applied, it requires the cooperation of relevant medical resources (such as doctor manpower and free wards) to produce the best configuration effect [27,28].
  It can be seen from the relevant literature that the allocation of nursing manpower in emergency room cases that can leave at any time is related to the patient’s care time and satisfaction. The correlation between less manpower allocation and poor outcomes is not clear. In particular, there is a lack of evidence regarding the impact of manpower allocation on direct patient outcomes, as well as a lack of sufficient economic analysis to provide information for decision-making on the allocation of nursing manpower. At present, it has been proved that there is a correlation between the level of allocation of nursing manpower and the outcomes of hospitalized patients, thus, more evidence is needed to understand nursing manpower allocation in emergency care [29].

## 6. Conclusions

This study mainly explored the waiting times of cases for emergency treatment and the nursing manpower allocation methods and adopted the queueing model theory to conduct a study on the triage of cases. The results show that the use of different priority beds for patients with different conditions and triage, based on different diagnoses and treatment methods, could effectively shorten the waiting times for medical treatment, examinations, and inspections. However, the difference in shortening the waiting time for nursing care is insignificant. Some factors causing the extended waiting time for nursing care in the emergency department could be insufficient nursing manpower planning and the retention of patients due to an insufficient number of hospital beds for patient transfer. The results of this study could provide decision-makers with a reference for using the queueing model theory to apply triage to cases in emergency departments in the future. The allocation and scheduling of nursing manpower could also be adjusted to improve the waiting time for emergency care.

How to maximize medical efficiency with the limited allocation of available resources is indeed a topic that needs to be carefully explored. In our report, we only considered that when the patient flow enters the emergency system, it automatically enters different flow lines according to the triage classification without further subdivision. In Taiwan, even the vast majority of patients at levels 4/5 still have to enter the queuing system as part of the emergency system, and they cannot be directly discharged from the system during inspection. In other words, while patients at levels 4/5 only have delayed access to the system, they will still occupy a portion of the system’s service time. In addition, this report did not conduct a comparison with the elementary stream flow, and only the recorded flow was statistically verified, according to the Poisson arrival rate.

Thus, the service scenario based on priority or other improvements has not yet been proposed in the report. In addition, the comparison and related derivation of the queueing model are still in progress in the form of a research project.

## Figures and Tables

**Table 1:** Change in waiting times caused by each one-minute reduction in reported wait times.

	Change	Waiting Time Value of Each Station		
		Medical Consultation	Inspection	Nursing
Surgical department	1	4.28	1.83	8.98
	2	3.86	1.56	8.83
	3	3.36	1.37	8.65
	4	2.78	1.32	8.26
	5	2.22	1.26	8.01
Internal medicine department	1	8.76	1.83	9.03
	2	8.32	1.75	8.87
	3	8.09	1.67	8.62
	4	7.84	1.58	8.42
	5	7.80	1.55	8.17
Pediatric department	1	2.01	1.83	6.34
	2	1.88	1.76	6.03
	3	1.73	1.64	5.82
	4	1.65	1.46	4.99
	5	1.57	1.25	4.76

Unit: minutes.

**Table 2:** Change in waiting time with each additional caregiver.

	Change	Waiting Time Value of Each Station		
		Medical Consultation	Inspection	Nursing
Surgical department	1	4.42	1.83	8.98
	2	3.67	1.32	8.72
	3	3.01	1.16	8.39
	4	2.36	1.10	8.11
	5	1.57	1.09	7.69
Internal medicine department	1	8.76	1.83	9.03
	2	8.28	1.63	8.62
	3	7.95	1.53	8.37
	4	7.76	1.42	8.15
	5	7.68	1.34	8.02
Pediatric department	1	2.01	1.83	6.34
	2	1.76	1.64	6.01
	3	1.54	1.41	5.64
	4	1.32	1.19	4.72
	5	1.09	1.02	4.53

Unit: minutes.

**Table 3:** Change in waiting time caused by each one-minute reduction in the doctor’s treatment time.

	Change	Waiting Time Value of Each Station		
		Medical Consultation	Inspection	Nursing
Surgical department	5	4.75	1.83	8.98
	6	4.42	1.34	8.65
	7	4.28	1.18	8.37
	8	4.03	1.08	8.16
	9	3.89	1.00	7.92
Internal medicine department	8	6.76	1.83	9.03
	9	6.54	1.63	8.55
	10	6.35	1.41	8.32
	11	6.18	1.25	8.04
	15	5.92	1.07	7.69
Pediatric department	2	1.01	1.83	6.34
	3	0.89	1.65	6.17
	4	0.76	1.39	5.98
	5	0.71	1.17	4.73
	6	0.54	1.05	4.38

Unit: minute.

**Table 4:** Results of burst entry simulated using Pareto distribution.

	Change α (k = 3.0)	Waiting Time Value of Each Station		
		Medical Consultation	Inspection	Nursing
Surgical department	1	4.58	1.83	8.98
	1.5	6.39	2.56	10.29
	2	8.32	4.38	13.75
	2.5	9.76	6.97	18.56
	3	11.35	9.84	22.83
Internal medicine department	1	8.76	1.83	9.03
	1.5	9.23	2.04	10.54
	2	9.98	3.16	11.97
	2.5	10.14	5.32	13.65
	3	12.56	7.74	14.82
Pediatric department	1	2.01	1.83	6.34
	1.5	2.98	2.45	7.02
	2	4.03	4.53	8.09
	2.5	5.78	6.97	9.28
	3	7.62	9.73	11.05

Unit: minutes.

**Table 5:** Effects of different dispersion probabilities.

	Change	Waiting Time Value of Each Station		
		Medical Consultation	Inspection	Nursing
Surgical department	0.1	4.65	1.83	8.98
	0.2	4.37	1.62	8.82
	0.3	4.15	1.51	8.53
	0.4	3.98	1.46	8.28
	0.5	3.76	1.43	8.02
Internal medicine department	0.1	8.76	1.83	9.03
	0.2	8.54	1.62	8.82
	0.3	8.36	1.48	8.76
	0.4	8.07	1.27	8.55
	0.5	7.86	1.19	8.38
Pediatric department	0.1	2.01	1.82	6.34
	0.2	1.93	1.80	6.26
	0.3	1.90	1.72	6.05
	0.4	1.87	1.57	5.81
	0.5	1.83	1.48	5.62

Unit: minutes.

## Data Availability

Data are contained within the article.
